# Nanoemulsification Enhances the Regenerative and Safety Profile of *Ocimum gratissimum* Essential Oil *In Vitro*

**DOI:** 10.1021/acsomega.5c05769

**Published:** 2025-09-28

**Authors:** Julia Salles Gava, Gabriela Aquino Simões, Danielle Braga Portes, Juliana Varela Cruz, Adriana Solange Maddaleno, Maria Pilar Vinardell, Montserrat Mitjans, Hildegardo Seibert França, Marcio Fronza

**Affiliations:** a Natural Products Laboratory, 125100 Vila Velha University, Av. Comissário José Dantas de Melo, 21, Vila Velha 29102-920, Brazil; b School of Pharmaceutical Sciences of Ribeirão Preto, São Paulo University, Av. Bandeirantes, São Paulo 14040-900, Brazil; c Department of Biochemistry and Physiology, 16724 Universitat de Barcelona, Av. Joan XXIII, 27-31, Barcelona 08028, Spain; d Bioproducts Development Laboratory, Federal Institute of Espírito Santo, Av. Min. Salgado Filho, Vila Velha 29106-010, Brazil

## Abstract

The essential oil of *Ocimum gratissimum* (OG) stands out as a multifunctional agent with antioxidant, anti-inflammatory, and antimicrobial properties, positioning it as a candidate for innovative cosmetic applications. However, its instability in aqueous formulations remains a key barrier to its practical use. Therefore, this study aimed to develop a nanoemulsion incorporating *Ocimum gratissimum* essential oil (NG) using a low-energy emulsification technique and comprehensively evaluate its biological properties. The chemical composition of OG was analyzed using gas chromatography–mass spectrometry (GC–MS). A series of *in vitro* cell-based assays and alternative testing methods were employed to evaluate the NG for its antioxidant and anti-inflammatory properties, cellular proliferation, migration, as well as cytotoxicity and overall safety. GC–MS analysis revealed β-ocimene, eugenol, and germacrene D as the major components of OG. The developed NG exhibited a particle size under 500 nm, low polydispersity index (<0.3), and negative zeta potential, maintaining physicochemical stability for up to 60 days at 4 °C. Although NG showed a lower antioxidant activity than OG in chemical assays (ABTS/DPPH), both formulations effectively reduced intracellular levels of superoxide, hydrogen peroxide, and nitric oxide in macrophages. Notably, NG inhibited TNF-α, IL-6, and IL-1 production, whereas OG only suppressed TNF-α. In fibroblast models, both NG and OG stimulated cell proliferation (BrdU assay) and migration (scratch assay). Safety evaluations confirmed low ocular irritability (HET-CAM and CAM-TBS) and no phototoxicity (NRU-3T3). These findings highlight that NG enhanced anti-inflammatory efficacy and biological compatibility and demonstrate its potential as a cosmetic ingredient for skin regeneration.

## Graphical abstract

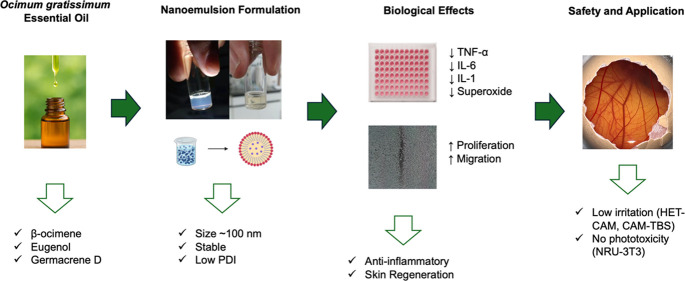

## Introduction

The increasing demand for natural, safe, and sustainable ingredients has encouraged the cosmetic and pharmaceutical industries to explore plant-based alternatives with proven bioactivity. Historically used by various civilizations, these plants offer bioactive compounds with well-recognized therapeutic efficacy, attracting increasing interest from modern science.^1^ Among the most valued of these compounds, essential oils stand out for their complex composition and diverse biological activities, including anti-inflammatory,^2^ antibacterial,^3^ and antioxidant^4^ properties.

Essential oils are volatile aromatic compounds extracted from plants, typically obtained by steam distillation or cold pressing, and are composed of a mixture of chemical groups such as monoterpenes, sesquiterpenes, and phenylpropanoids, which are responsible for their characteristic aroma and bioactivity. Despite their benefits, these compounds are often chemically unstable, being susceptible to oxidation, volatilization, and degradation when exposed to light, heat, or oxygen.^5^

Their functional versatility makes them particularly attractive for innovative cosmetic formulations aligned with green cosmetic trends.^6^ Within this context, Brazil, known for its rich biodiversity, represents one of the main global sources for research and development of natural actives, reinforcing its strategic role in biotechnology and the valorization of native plant resources.^7^

Among the species of interest, *Ocimum gratissimum* stands out for its essential oil rich in compounds with antioxidant,^8,9^ analgesic,^10^ antifungal,^11,12^ antimicrobial,^11,13–15^ insecticidal,^16^ and antimelanosis^17^ properties, which have drawn increasing attention from the scientific community. However, the instability of essential oils in aqueous vehicles commonly used in cosmetics limits their therapeutic potential.^18^

Nanoemulsions, defined as kinetically stable colloidal dispersions of oil and water stabilized by surfactants, have emerged as an innovative strategy to overcome these limitations. They feature droplet sizes in the nanometric range (typically 20–200 nm), which reduce gravitational separation and creaming, and can be stabilized by either steric or electrostatic mechanisms. Nevertheless, their stability can still be affected by phenomena such as Ostwald ripening, coalescence, and phase separation, especially under temperature fluctuations or inadequate surfactant concentration.^19^

To overcome these limitations, nanoemulsions have emerged as an innovative solution to preserve and enhance these properties, allowing for a safer and more effective application. By reducing the droplet size of essential oils to the nanometric scale, nanoemulsions offer improved physical stability and bioavailability, making them suitable for aqueous cosmetic formulations.^20^ This approach not only increases the efficacy of bioactive compounds but also expands the potential uses of this plant in cosmetic and pharmaceutical products.^21^ Moreover, nanoemulsions are known for their capacity to penetrate deeper skin layers, enhancing antioxidant and anti-inflammatory effects, which can be particularly beneficial in topical applications.^22^

In this scenario, the safety assessment of formulations such as nanoemulsion-based systems requires more precise and ethical methods. With scientific and regulatory advancements, alternative methods to animal testing have assumed prominent roles and acceptance in the evaluation of cosmetic ingredients. Based on the 3R’s principle—Replace, Reduce, and Refine—proposed by William Russell and Rex Burch in 1959, there has been a growing need to develop validated alternative models, promoting further investigation in this field.^23^ The implementation of these methods follows international guidelines such as those established by the OECD, ensuring validity and reproducibility. In Brazil, ANVISA and CONCEA have played a key role in regulating and encouraging the use of these approaches, promoting the progressive replacement of animal testing without compromising product safety and efficacy.^24^

Therefore, the aim of this study was to develop and characterize a nanoemulsion containing *Ocimum gratissimum* essential oil (NG), evaluating its safety profile and *in vitro* biological activities with an emphasis on antioxidant, anti-inflammatory, and wound healing properties. The proposed investigation not only contributes to the advancement of nanoemulsions as efficient delivery systems for bioactive compounds but also offers new perspectives for the use of *Ocimum gratissimum* in cosmetic formulations. It is believed that this approach may significantly improve the efficacy and safety of these products and open new avenues for applications in the cosmetic field.

## Material and Methods

### Materials

Polysorbate 80 (Tween 80) was purchased from Sigma-Aldrich (St. Louis, MO, USA). Sodium chloride (NaCl), phosphate-buffered saline (PBS, pH 7.4), sodium dodecyl sulfate (SDS, 1%), and sodium hydroxide (NaOH, 0.1 N) were obtained from Merck (Darmstadt, Germany). Dimethyl sulfoxide (DMSO), 3-(4,5-dimethylthiazol-2-yl)-2,5-diphenyltetrazolium bromide (MTT), neutral red dye (NRU), 2,2-diphenyl-1-picrylhydrazyl (DPPH), and 2,2’-azino-bis­(3-ethylbenzothiazoline-6-sulfonic acid) (ABTS) were acquired from Sigma-Aldrich. The essential oil of *Ocimum gratissimum* (OG) was supplied by the HAJE Company, certified, and stored in amber bottles under refrigeration (4 °C). The fibroblast cell line L929 (ATCC CRL-6364), 3T3, RAW 264.7 macrophage cell line (ATCC TIB-71), and HaCaT keratinocytes were cultured in Dulbecco’s modified Eagle’s medium (DMEM) suitable for each cell type, supplemented with 10% fetal bovine serum, l-glutamine (when necessary), and penicillin–streptomycin at varying concentrations. Cells were maintained at 37 °C in a humidified atmosphere with 5% CO_2_ and 97% relative humidity until reaching 80% confluence. The 3T3 (Balb/3T3 clone A31) cell line was acquired from Cytion (CLS Cell Lines Service GmbH, Germany). HaCaT was acquired from Eucellbank (Celltec UB, University of Barcelona, Spain), while the others were obtained from well-established collections such as ATCC.

### Chemical Characterization of *Ocimum gratissimum* Essential Oil by Gas Chromatography Coupled to Mass Spectrometry (GC–MS)

For the chemical characterization of OG, analyses were performed using a gas chromatograph coupled to a mass spectrometer (Agilent 7890 B) with a 5977A MSD mass detector operating with 70 eV electron impact ionization. An HP-5 column (30 m × 250 μm × 0.25 μm) was used for the separation process. The injector temperature was set to 290 °C and the detector to 310 °C. The elution system followed a temperature gradient starting at 40 °C, with a heating rate of 5 °C/min up to 280 °C followed by a heating rate of 15 °C/min up to 310 °C. For compound characterization, an *n*-alkane standard ranging from C10 to C40 was used to calculate the Kovats Retention Index (KI). Compound identification was carried out by comparing the mass spectra obtained with those in the NIST library, along with retention indices and mass spectral similarity with literature data.^25^

### Formulation of Nanoemulsions

Nanoemulsions containing 5% (w/w) OG essential oil were prepared using a low-energy emulsification method.^26^ Various combinations and concentrations of emulsifiers, including polysorbate 20, polysorbate 80, sorbitan monooleate, and sorbitan trioleate, were tested at concentrations ranging from 5 to 20%, adjusting the hydrophilic–lipophilic balance (HLB) values to 10, 11, 15, and 20. The oil phase, composed of the essential oil and selected emulsifiers, was mixed with the aqueous phase under vortex agitation to form nanostructured colloidal systems. From the 5% system, serial dilutions in water were performed to obtain final concentrations of 2.5, 1, 0.5, and 0.25. All formulations were subjected to stability tests at 8 and 40 °C and centrifuged at 3000 rpm for 30 min. The most macroscopically stable systems were selected for further analysis. Approved samples were then evaluated for pH, refractive index, and transmittance at 600 nm.^27^

### Characterization of the Nanoemulsions

#### Determination of Particle Size, Polydispersity Index, and Zeta Potential (ζ)

The particle size (nm), polydispersity index (PDI), and zeta potential (mV) of *Ocimum gratissimum* nanoemulsions (NGs) were determined on days 1 (D1), 7 (D7), 15 (D15), 30 (D30), and 60 (D60) by dynamic light scattering (DLS) using a 658 nm laser and scattering angles of 15, 90, and 175° with a Litesizer 500 instrument (Anton Paar, Graz, Austria). The parameters were estimated based on the average of three measurements. This methodology was adapted from Ontao et al.^28^

#### Stability at Different Storage Temperatures

The thermal stability of the *Ocimum gratissimum* nanoemulsions (NGs) was evaluated by particle size (nm), polydispersity index (PDI), and zeta potential (mV) by dynamic light scattering (DLS) on D1, D7, D15, D30, and D60. The nanoemulsions were aliquoted in triplicate and stored under three different conditions: 4 ± 2, 25 ± 2, and 37 ± 2 °C.^29^

### Antioxidant Activity

The antioxidant activity of NG and OG was assessed using the ABTS (2,2’-azino-bis­(3-ethylbenzothiazoline-6-sulfonic acid)) and DPPH (2,2-diphenyl-1-picrylhydrazyl) radical scavenging assays.^30^ For both assays, samples were prepared at concentrations ranging from 0.1 to 100 μg/mL in 70% ethanol and were subsequently diluted directly in microplates. The plates were kept in the dark at room temperature for 60 min before absorbance readings at 517 nm (DPPH) and 734 nm (ABTS). Results were expressed as IR_50_, representing the concentration required to inhibit 50% of free radicals. All experiments were performed in triplicate.

### *In Vitro* Alternative Safety Methods for NG

#### Photoprotective Activity

The procedure was approved by the Bioethics Committee at the University of Barcelona, Spain (11 January 11, 2024). A total of 5 mL of blood from a healthy human donor was collected into an anticoagulant solution containing EDTA. Erythrocytes were then isolated by centrifugation at 3000 rpm for 10 min and washed three times with isotonic phosphate-buffered saline (PBS) at pH 7.4.^31^ The method used was an adaptation described by Pape et al. (2001).^32^ NG at 30 μg/mL was added to 25 μL aliquots of the erythrocyte suspension in 24-well plates and irradiated for 150 min with a UV dose of 15 J/cm^2^ UVA using Actinic BL 15W/10 FAM/10X25BOX lamps (Royal Philips Electronics, The Netherlands) with a 315–400 nm spectral range.^33^ The lamps’ energy output was periodically checked before each experiment using a Delta OHM photoradiometer provided with a UVA probe (HD2302, Italy). Dark controls were incubated simultaneously in the UV cabin but preserved from irradiation. This period was followed by a 30 min incubation in the dark before measurements were taken. Samples were then centrifuged at 10,000 rpm for 5 min. Supernatants were transferred to cuvettes, and the degree of hemolysis was measured by absorbance at 525 nm by using a Shimadzu UV–vis 160A dual-beam spectrophotometer (Kyoto, Japan). Hemolysis percentages were determined by comparison with hemolyzed red blood cells (RBCs) with distilled water, and positive controls consisted of RBCs treated with the well-known photosensitizer chlorpromazine (CPZ) at 60 μg/mL. In parallel, the oxidation of hemoglobin was also determined at 630 nm after total lysis of the erythrocytes with Triton-X 100.

#### Evaluation of Ocular Irritation Potential by the Hen’s Egg Chorioallantoic Membrane (HET-CAM) Test

The HET-CAM test was used to evaluate the irritant potential of the formulations on the chorioallantoic membrane (CAM). Fertilized White Leghorn chicken eggs, acquired from a hatchery in Espírito Santo, Brazil, were inspected and incubated for 10 days at 37.5 °C and 62.5% humidity. On the 10th day, the eggshell was carefully removed around the air chamber to expose the CAM, which was hydrated with saline solution for 1 min to facilitate exposure without damage.^34^ For each membrane, 300 μL of NG was applied and maintained for 20 s before being rinsed with 5 mL of 0.9% saline solution at 37 °C. The procedure was repeated for the positive control (0.1 N NaOH) and the negative control (0.9% saline). After removal, the CAM was observed for 5 min to identify irritative reactions such as hemorrhage and coagulation, which were classified based on their onset time and irritation score.^35^

#### Evaluation of Ocular Irritation Potential by the Chorioallantoic Membrane-Trypan Blue Staining (CAM-TBS) Test

CAM-TBS is a quantitative method for evaluating the toxicity of formulations. The CAM-TBS test follows procedures similar to the HET-CAM method; however, it uses trypan blue as an indicator of injury to the CAM. After 10 days of incubation, the eggs were positioned with the air chamber facing upward. The eggshell and the whitish inner membrane were carefully removed to expose the CAM, which was delimited using a silicone ring.^35^ Samples (0.3 mL in 0.9% saline solution) were applied for 20 s and then removed. Next, 0.1% trypan blue was added, and the excess dye was rinsed off. The delimited area was excised, placed in formamide, and centrifuged to extract the dye. The absorbance of the supernatant was measured by spectrophotometry at a wavelength of 610 nm using test tubes containing 5 mL of formamide. The amount of trypan blue adsorbed in the chorioallantoic membrane was calculated.

#### *In Vitro* Phototoxicity Test on 3T3 Fibroblasts Using Neutral Red Uptake (NRU)

The phototoxicity evaluation followed the guidelines of the Organization for Economic Co-operation and Development with some adaptations.^36^ Fibroblasts of the 3T3 cell line were cultured for 24 h to form monolayers. Two 96-well plates per cell line were preincubated in a serum-free, phenol-red-free medium for 1 h. One plate for each cell line was exposed to a UVA radiation dose of 4 J/cm^2^, while the other was kept in the dark. UVA irradiation was performed using previous lamps described, and the exposure time was determined using the following formula:

$$E(J/{\mathrm{cm}}^{2})=T(s)\times P(W/{\mathrm{cm}}^{2})$$ 1

where *E* is the UV dose, *T* is the time in seconds, and *P* is the lamp power. After exposure, the treatment medium was replaced with culture medium, and after 24 h, cell viability was determined using the neutral red uptake (NRU) assay. Absorbance was measured at 550 nm after 3 h of incubation using a Tecan microplate reader. To predict phototoxic potential, cell viabilities obtained in the presence and absence of UVA radiation were compared.^36^

### *In Vitro* Assays

#### *In Vitro* Cytotoxicity Evaluation

Cell viability of the nanoemulsion (NG) and the essential oil (OG) was assessed using the colorimetric MTT assay (3-(4,5-dimethylthiazol-2-yl)-2,5-diphenyltetrazolium bromide)^37^ and the neutral red uptake (NRU) assay.^38^ L929 fibroblasts, RAW 264.7 macrophages, and HaCaT keratinocytes were exposed to different sample concentrations (20, 10, 5, 2.5, and 1.25 μg/mL) for 24 h. The assays were performed in triplicate, and the results were expressed as the percentage of cell viability with respect to untreated or control cells.

#### Preventive Effect against Oxidative Damage in RAW 264.7 Caused by H_2_O_2_

The protective effect of OG and NG against oxidative damage caused by hydrogen peroxide (H_2_O_2_) in RAW 264.7 macrophages was evaluated according to a method adapted by de Souza Júnior et al.^30^ Cells were seeded in 96-well plates at a density of 7 × 10^4^ cells/mL. After 24 h, cells were exposed to different concentrations of OG and NG, and 500 μM of H_2_O_2_ was added. After 24 h of incubation, cell viability was assessed using the MTT assay. Catalase (CAT) was used as a positive control. Experiments were performed in triplicate, and results were expressed as the mean ± standard deviation (SD) of cell viability (%).

#### *In Vitro* Indirect Determination of Nitric Oxide

Indirect quantification of nitric oxide (NO) was carried out by determining the influence on nitrite production in lipopolysaccharide (LPS)-activated RAW 264.7 macrophage culture, as described by de Souza Júnior et al.^30^ Macrophages were cultured in 96-well plates at a concentration of 10 × 10^4^ cells/mL for 24 h in a 5% CO_2_ atmosphere at 37 °C. Samples were then exposed to different concentrations of NG and stimulated with 1 μg/mL LPS. After 24 h, the supernatant was collected for nitrite quantification using the Griess reagent (1% sulfanilamide in 5% phosphoric acid and 0.1% *N*-(1-naphthyl)­ethylenediamine dihydrochloride in water; 1:1)^38^ Absorbance was read at 540 nm using a microplate reader, and results were expressed as the mean nitrite concentration in micromolars. Experiments were conducted in triplicate, and results were expressed as mean ± SD.

#### Superoxide Anion Production Reduction

RAW 264.7 macrophages, at a concentration of 10 × 10^4^ cells/mL, were cultured in 96-well plates and stimulated with LPS according to de Souza Júnior et al.^30^ After exposure to different sample concentrations for 24 h, superoxide anion (O_2_^–^) production was assessed. A concentration of 10 μM l-NAME was used as a positive control. The supernatant was removed, and cells were exposed to NBT (1 mg/mL) for 2 h. Cells were then washed with methanol, and the resulting formazan crystals were dissolved in a 2 M KOH and DMSO solution. Absorbance was measured at 630 nm, and the results were expressed as the percentage of the production of O_2_^–^ production. Experiments were conducted in triplicate, and results were expressed as mean ± SD.

#### *In Vitro* Cytokine Determination

Quantification of TNF-α and IL-6 cytokines in RAW 264.7 macrophage cultures stimulated with 1 μg/mL LPS and exposed to various sample concentrations was performed using an enzyme-linked immunosorbent assay (ELISA). The procedure used specific antibodies and standards for each cytokine according to the manufacturer’s instructions (Invitrogen). Readings were taken at 450 nm by using a microplate reader. Experiments were conducted in triplicate, and the results were expressed in picograms per milliliter (pg/mL).

#### Cell Proliferative Activity (BrdU)

The effect on fibroblast proliferation was determined using the Cell Proliferation Quantification Kit-BrDU according to the manufacturer’s specifications. Fibroblast cells were cultured in Dulbecco’s modified Eagle’s medium (DMEM) supplemented with 10% fetal bovine serum (FBS) at 37 °C and 5% CO_2_. Cells were seeded in 96-well plates at a density of 3 × 10^3^ cells/90 μL/well of the serum-free medium. After 24 h, the proliferative effect of NG at different concentrations was measured using the thymidine analogue BrdU (5-bromo-2’-deoxyuridine) after incorporation into newly synthesized DNA and detection using an anti-BrdU antibody according to the manufacturer’s instructions (Roche Mannheim, Germany).

#### Cell Migration (Scratch Assay)

The scratch assay was conducted to evaluate *in vitro* wound healing stimulation as described by Fronza et al. (2009).^40^ Cells were cultured in six-well plates until reaching 90–100% confluence. Linear scratches were made in the monolayers using a sterile 100 μL pipet tip, simulating wounds. NG was tested at a concentration of 20 μg/mL. After treatment, cells were fixed with paraformaldehyde. Cell migration was recorded by microscopy, and the results were expressed as the percentage of cell proliferation and migration into the wounded area.

#### Tyrosinase Inhibition Assay

The skin-whitening effect of OG was evaluated using a modified tyrosinase inhibition method as previously described.^41^ A solution of tyrosine (120 μL of 1.66 mM in 0.1 M phosphate buffer, pH 6.8) was added to 60 μL of OG to obtain final concentrations of 10, 1, and 0.1 μg/mL, along with 60 μL of phosphate buffer in a 96-well plate. The mixture was incubated at 37 ± 2 °C for 20 min. Then, 60 μL of a tyrosinase enzyme solution (150 mM in phosphate buffer) was added. Enzymatic activity was measured at 37 ± 2 °C using a microplate reader at 450 nm. Kojic acid (125 μg/mL) was used as a positive control. Experiments were conducted in triplicate. The IC_50_ value, representing the sample concentration required to inhibit 50% of enzymatic activity, was determined. The percentage of tyrosinase inhibition was calculated using the following formula:

inhibition(%)=[(abscontrol−abssample)/abscontrol]×100% 2

### Statistical Analysis of Data

Statistical analysis was performed using GraphPad Prism version 10.00. IC_50_ values with a 95% confidence interval were determined by nonlinear regression. Statistical differences between IC_50_ values were calculated using one-way analysis of variance (ANOVA) followed by Tukey’s *post hoc* test for multiple comparisons. *p* values < 0.05 were considered statistically significant.

## Results

### Chemical Composition of Essential Oil

The compounds present in the essential oil of *Ocimum gratissimum*, identified by gas chromatography coupled with mass spectrometry (GC–MS), are detailed in Table 1. The corresponding chromatogram is shown in Figure 1. Among the identified compounds, the major constituents were β-ocimene (24%), eugenol (21%), and germacrene D (14.5%).

**Figure 1:**
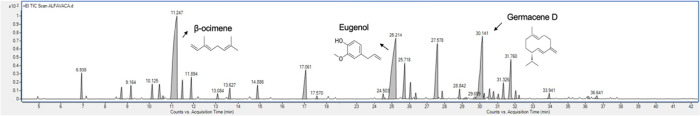
Chromatogram of *Ocimum gratissimum* essential oil.

**Table 1:** Chemical Composition of *Ocimum gratissimum* Essential Oil^a^

identification	*T*_r_	IR_calc_	IR_lit_	% area
thujene	6.938	926	924	1.671
1-octen-3-ol	8.741	980	974	1.007
myrcene	9.164	992	988	1.005
terpinene α→	10.125	1017	1014	1.243
cymene < ο→	10.452	1025	1022	1.373
**ocimene < (e)-β→**	**11.247**	**1044**	**1044**	**24.270**
ocimene < (z)-β→	11.493	1050	1032	1.331
terpinene < γ→	11.894	1060	1054	1.679
terpinolene	13.084	1089	1086	0.441
linalool	13.627	1102	1095	0.975
ocimene < allo→	14.886	1130	1128	1.243
terpinen-4-ol	17.061	1179	1174	4.068
terpineol < α→	17.570	1191	1186	0.261
cubebene < α→	24.503	1350	1348	0.597
**eugenol**	**25.214**	**1366**	**1356**	**21.010**
copaene < α→	25.718	1378	1374	4.395
bourbonene < β→	26.056	1386	1387	1.588
elemene < β→	26.353	1393	1389	0.539
caryophyllene < (e)→	27.578	1423	1417	9.705
copaene < β→	27.858	1430	1430	0.717
humulene < α→	28.842	1454	1452	0.922
**germacrene D**	**30.141**	**1486**	**1480**	**14.596**
selinene < β→	30.244	1488	1489	0.420
muurolene < α→	30.771	1501	1500	0.739
cadinene < γ→	31.326	1516	1513	1.653
cadinene < δ→	31.760	1527	1522	5.126
cadinene < α→	32.224	1539	1537	0.316
caryophyllene oxide	33.941	1583	1582	0.563
muurolol < epi-α→	36.184	1643	1640	0.274

a *T*_r_ = retention time (min); IR_calc_ = calculated retention index; IR_lit_ = literature retention index; and % area = relative concentration of each compound in relation to the total chromatogram area.

### Preparation of the Nanoemulsion

After extensive analysis of all formulation variations, the most macroscopically stable dilution was the 1% formulation. The optimal result was achieved using polysorbate 80 with an HLB value of 15, at a ratio of 5% essential oil, 15% emulsifier, and 80% water. Under these conditions, the nanoemulsions exhibited favorable characteristics, including the absence of phase separation, slight viscosity, translucency, and a bluish hue when exposed to direct light. Moreover, stability tests confirmed the absence of creaming or breakdown even under temperature fluctuations, highlighting the robustness of this formulation. The final concentration of 1% was selected for subsequent analyses in the study.

### Temporal and Thermal Stability

The physical stability of NG was evaluated at different temperatures (4, 25, and 45 °C) over a period of 60 days, considering parameters such as average particle size, zeta potential, and polydispersity index (PDI), as presented in Table 2 and Figure 2. Representative images showing the visual appearance of the nanoemulsion containing *Ocimum gratissimum* essential oil before (D0) and after 60 days (D60) of storage under these temperature conditions are provided as Supporting Information (Figures S1 and S2).

**Table 2:** Analysis of Particle Size (nm), Zeta Potential (mV), and Polydispersity Index (PDI) of NG over 60 Days (D0 to D60) as Determined by DLS at Temperatures of 4, 25, and 45°C^a^

	average size (nm)			zeta potential (mV)			PDI (%)		
	**4 °C**	**25 °C**	**45 °C**	**4 °C**	**25 °C**	**45 °C**	**4 °C**	**25 °C**	**45 °C**
D0	112 ± 0.9^a.c^	146.3 ± 4.7^a^	204.8 ± 4.8^a^	–5.2 ± 0.1^a^	–4.1 ± 0.7^a^	–5.2 ± 0.5^a^	0.2 ± 0.0^a^	0.2 ± 0.0^a^	0.2 ± 0.0^a^
D7	101.4 ± 0.2^a^	214.5 ± 2.4^b^	340 ± 14.5^b^	–4.2 ± 0.2^a^	–2.9 ± 1.0^a.b^	–5.1 ± 1.0^a.b^	0.2 ± 0.0^a^	0.2 ± 0.0^a^	0.2 ± 0.0^a^
D15	71.4 ± 1.9^b^	265.0 ± 3.7^c^	290 ± 3.4^b^	–3.7 ± 0.1^a^	–1.9 ± 1.2^b^	–4.63 ± 0.1^b^	0.2 ± 0.0^a^	0.2 ± 0.0^a^	0.2 ± 0.0^a^
D30	118.0 ± 0.2^c^	340.2 ± 9.3^d^	247.8 ± 6.3^d^	–2.3 ± 0.3^b^	–1.2 ± 0.43^c^	–3.49 ± 1.3^b^	0.2 ± 0.0^a^	0.2 ± 0.0^a^	0.2 ± 0.0^a^
D60	101.0 ± 1.1^a^	333.3 ± 4.5^d^	50 ± 4.5^e^	–4.1 ± 0.4^a.c^	–2.0 ± 0.5^a.b^	–4.86 ± 0.5^c^	0.2 ± 0.0^a^	0.2 ± 0.0^a^	0.2 ± 0.0^a^

a Different letters in the same column indicate significant differences between samples (*p* < 0.05). Tests were performed in triplicate, and results are presented as mean ± SD.

**Figure 2:**
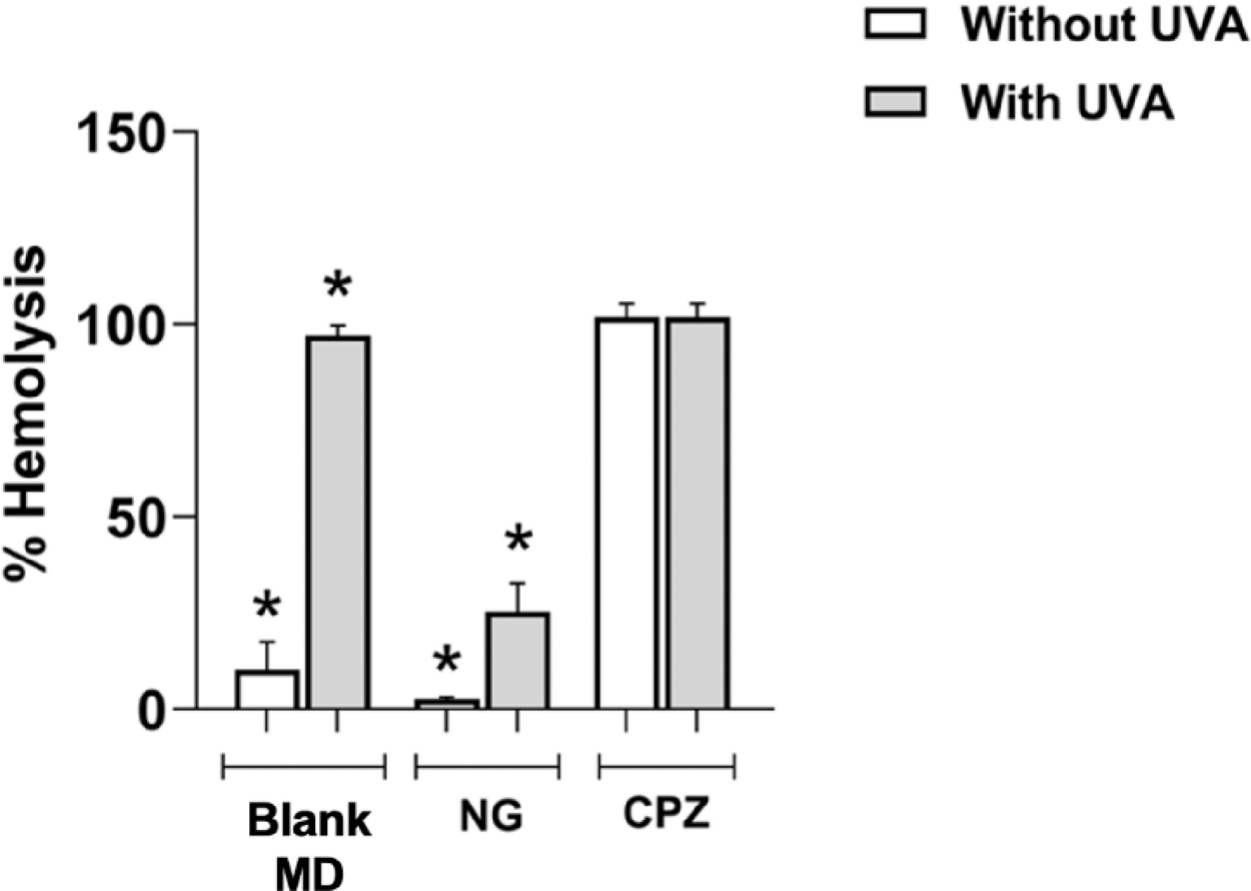
Photohemolysis of *Ocimum gratissimum* nanoemulsion (NG) under UVA exposure (gray bars) and in the absence of UVA exposure (white bars) at 15 J/cm^2^. Hemolysis was evaluated for NG (30 μg/mL), micellar dispersion formed by emulsifier plus water in the same concentration of NG without essential oil (blank MD), and chlorpromazine (CPZ, 60 μg/mL, positive control). Results are presented as the mean ± SD from three experiments performed in triplicate. Statistical analysis was performed using ANOVA followed by Tukey’s test.

### Antioxidant Activity

The antioxidant activity of OG, NG, and eugenol was evaluated using the DPPH and ABTS radical scavenging methods. Eugenol, one of the major identified compounds, was also assessed. The results are presented in Table 3.

**Table 3:** Antioxidant Activity Determined by ABTS and DPPH Assays of the *Ocimum gratissimum* Nanoemulsion (NG), *Ocimum gratissimum* Essential Oil (OG), and Eugenol Expressed as IR_50_ ^a^

	IR_50_ (μg/mL)	
**samples**	**ABTS**	**DPPH**
NG	3.14 ± 0.01^a^	0.12 ± 0.01^a^
OG	1.06 ± 0.12^b^	0.11 ± 0.01^a^
eugenol	1.36 ± 0.34^b^	3,05 ± 0.10^b^

a Results are presented as mean ± standard deviation. Different letters indicate statistically significant differences between samples (*p* < 0.05).

### *In Vitro* Alternative Safety Methods for NG

#### Photoprotective Activity

NG showed a considerably reduced percentage of hemolysis compared to the positive controls in both the absence and presence of UVA radiation. The blank MD group demonstrated high levels of hemolysis under UVA exposure. UVA exposure increased hemolysis induced by NG, but the values remained below those observed for chlorpromazine (CPZ), which exhibited a marked hemolytic effect (Figure 2).

For control purposes in photoprotective activity, HET-CAM, and CAM-TBS assays, a blank micellar dispersion (blank MD) was prepared using the same surfactant concentration (polysorbate 80) and aqueous phase as the nanoemulsion but without the addition of *Ocimum gratissimum* essential oil. In the absence of the oil phase, this system is technically a micellar dispersion rather than a nanoemulsion.

#### Evaluation of Ocular Irritation Potential by the HET-CAM Assay

The HET-CAM assay was performed to determine the ocular irritation potential of the NG. Table 4 presents the mean cumulative scores obtained as well as the positive controls (NaOH and SDS) and the negative control (0.9% NaCl).

**Table 4:** Effects of the Nanoemulsion of *Ocimum gratissimum* (NG) on the Ocular Irritation Potential Assessed by the HET-CAM Assay^a^

**samples**	**classification**
NG	irritant
blank MD	irritant
NaCl 0.9%	nonirritant
SDS 1%	severe irritant
NaOH 0.1 N	severe irritant

a The control formulation with micellar dispersion formed by emulsifier plus water in the same concentration of NG without essential oil (blank MD), 0.9% NaCl physiological solution (negative control), and 1% SDS and 0.1 N NaOH (positive controls) were also evaluated for ocular irritancy.

The HET-CAM assay was validated as appropriate since the 0.9% NaCl solution (negative control) was classified as “nonirritant” to the chorioallantoic membrane. On the other hand, the positive controls, 1% SDS and 0.1 N NaOH, were classified as “severe irritant” under the tested conditions, confirming the reliability of the method.

#### Evaluation of Ocular Irritation Potential by the CAM–TBS Assay

The semiquantitative evaluation of the ocular irritation potential of NG, determined by the CAM-TBS assay, showed no irritating effects, as demonstrated in Table 5.

**Table 5:** Effects of the *Ocimum gratissimum* Nanoemulsion (NG) on the Ocular Irritation Potential Assessed by the CAM-TBS Assay^a^

**samples**	**classification**
NG	nonirritant/mild irritant
blank MD	nonirritant/mild irritant
SF 0.9%	nonirritant/mild irritant
SDS 1%	severe irritant
NaOH 0.1 N	severe irritant

a The control formulation with micellar dispersion formed by emulsifier plus water in the same concentration of NG without essential oil (blank MD), 0.9% NaCl physiological solution (negative control), and 1% SDS and 0.1 N NaOH (positive controls) were also evaluated for ocular irritancy.

#### *In Vitro* Phototoxicity Test on 3T3 Fibroblasts Using Neutral Red Uptake (NRU)

The phototoxicity of the nanoemulsion was evaluated in mouse 3T3 fibroblasts using the neutral red uptake (NRU) assay. The results indicated that NG did not exhibit phototoxic effects at the tested concentrations (1 and 10 μg/mL), either with or without UVA exposure (4 J/cm^2^) (Figure 3).

**Figure 3:**
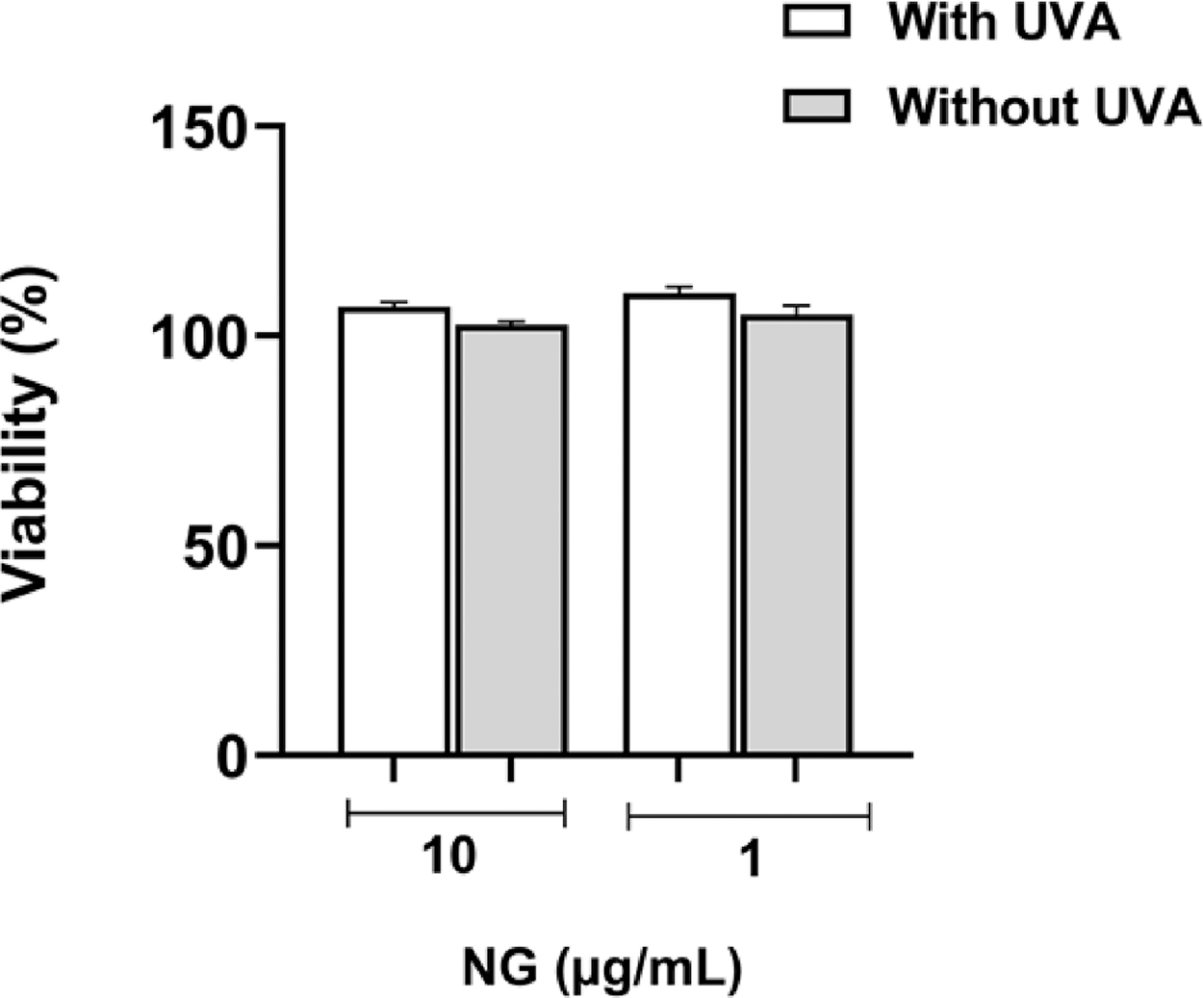
Phototoxic effect of *Ocimum gratissimum* nanoemulsion (NG) on 3T3 fibroblasts with and without UVA exposure (4 J/cm^2^). Cell viability was assessed using the neutral red uptake (NRU) assay after treatment with NG at concentrations of 1 and 10 μg/mL.

### *In Vitro* Assays

#### Effects of the Nanoemulsion Viability on the Cell Lines

The effects of NG and OG on *in vitro* cell viability using the L929 fibroblast, RAW 264.7 macrophage, and HaCaT keratinocyte cell lines were evaluated. The results revealed that the nanoemulsion at the various tested concentrations (20, 10, 5, 2.5, and 1.25 μg/mL) did not show cytotoxic effects on the tested cell lines with a cell viability higher than 90%. The blank NG was also tested under the same conditions and did not exhibit any cytotoxic effects.

#### Protective Effect against *In Vitro* Oxidative Damage Induced by H_2_O_2_

The cell viability of RAW 264.7 macrophages treated with NG and OG after exposure to hydrogen peroxide (H_2_O_2_) is shown in Figure 4. The results demonstrate that both OG and NG, at concentrations of 1 and 10 μg/mL, exhibited protective effects on the viability of RAW 264.7 macrophages under oxidative stress conditions induced by H_2_O_2_.

**Figure 4:**
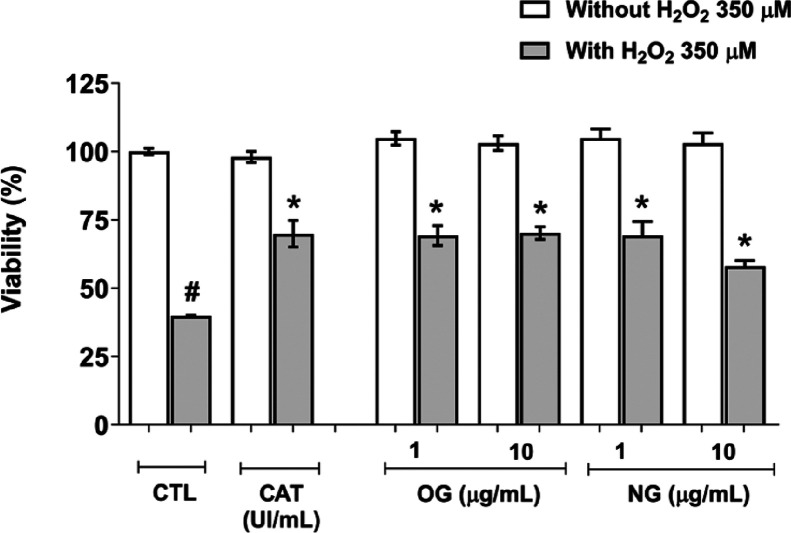
Protective effect of nanoemulsion (NG) and essential oil (OG) in RAW 264.7 macrophages against damage caused by hydrogen peroxide (H_2_O_2_). CTL indicates the treated control group. Catalase at 10 IU/mL (CAT) was used as positive control. Results are expressed as mean ± SD. The pound symbol (#) indicates significant difference (*p* < 0.05) compared to the negative control without H_2_O_2_. The asterisk (*) indicates significant difference (*p* < 0.05) compared to cells exposed only to H_2_O_2_ by one-way ANOVA followed by Tukey’s *post hoc* test.

#### *In Vitro* Indirect Determination of Nitric Oxide

Nitric oxide (NO) production was evaluated in macrophages stimulated with lipopolysaccharide (LPS), a strategy commonly used to induce inflammatory responses (Figure 5). As a result, the different concentrations of NG did not show a significant effect on NO production in macrophages activated with 1 μg/mL LPS. l-NAME 10 μM was used as a positive control.

**Figure 5:**
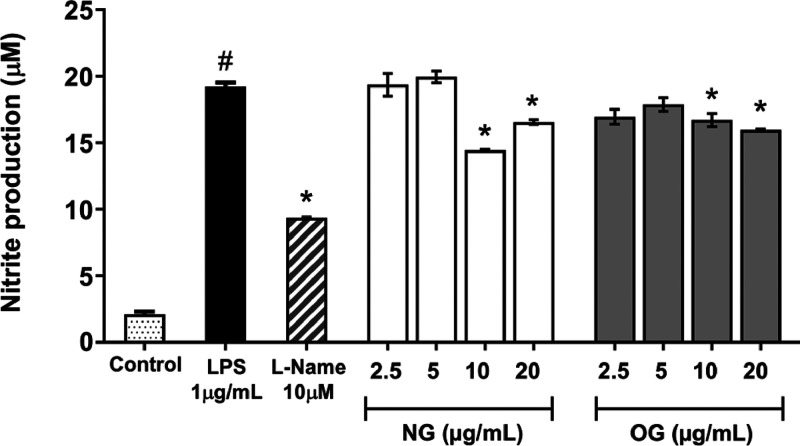
Effect of nanoemulsion (NG) and essential oil (OG) on nitrite production. RAW 264.7 macrophages were exposed to different sample concentrations and stimulated with LPS (lipopolysaccharide) (1 μg/mL). l-NAME at 10 μM was used as a positive control. The results were expressed as mean ± SD (*n* = 2). The pound symbol (#) indicates significant difference (*p* < 0.05) compared to the negative control without LPS. The asterisk (*) indicates significant difference (*p* < 0.05) compared to LPS-treated cells by one-way ANOVA followed by Tukey’s *post hoc* test.

#### Superoxide Anion Production Reduction

The results presented in Figure 6 demonstrate a dose-dependent inhibitory effect of both NG and OG on superoxide anion production in LPS-stimulated RAW 264.7 macrophages.

**Figure 6:**
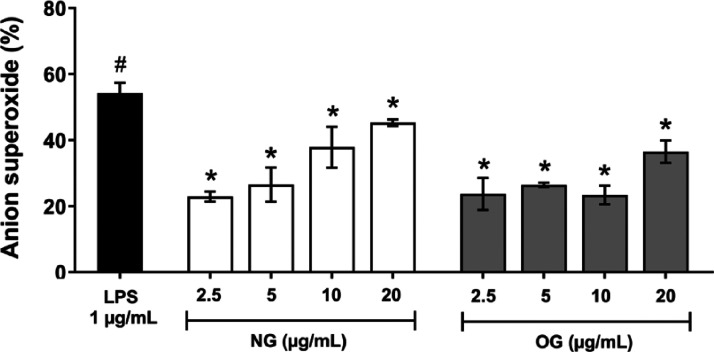
Inhibitory effects of nanoemulsion (NG) and essential oil (OG) on superoxide anion production. RAW 264.7 macrophages were exposed to different sample concentrations and stimulated with LPS (lipopolysaccharide) (1 μg/mL). The results were expressed as mean ± SD (*n* = 2). The pound symbol (#) indicates significant difference (*p* < 0.05) compared to the negative control without LPS. The asterisk (*) indicates significant difference (*p* < 0.05) compared to LPS-treated cells by one-way ANOVA followed by Tukey’s *post hoc* test.

#### *In Vitro* Cytokine Determination

The results presented in Figure 7 show the effects of NG and OG samples at concentrations of 2.5 and 5 μg/mL on the production of proinflammatory cytokines (IL-6, TNF-α, and IL-1) in LPS-stimulated RAW 264.7 macrophages. While OG was able to inhibit only TNF-α production at 5 μg/mL, NG showed an inhibitory effect on the production of IL-6, TNF-α, and IL-1 at the same concentration.

**Figure 7:**
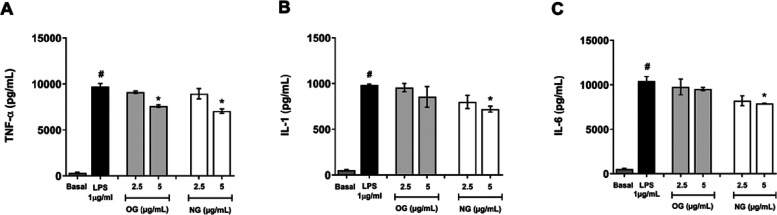
Effect of nanoemulsion (NG) and essential oil (OG) at concentrations of 2.5 and 5 μg/mL on the levels of the proinflammatory cytokines TNF-α (A), IL-1 (B), and IL-6 (C) in RAW 264.7 macrophage cultures stimulated with lipopolysaccharide (LPS, 1 μg/mL) after 24 h of treatment. Results are expressed as mean ± SD. Statistical significance was set at *p* < 0.05. Pound symbol (#): basal control group compared to LPS-stimulated group. Asterisk (*): treated group compared to LPS group.

#### Cell Proliferative Activity (BrdU)

The results shown in Figure 8 demonstrate the effect on cell proliferation as assessed by the BrdU assay. Both NG and OG significantly increased the proliferative activity of fibroblasts at concentrations of 10 and 20 μg/mL.

**Figure 8:**
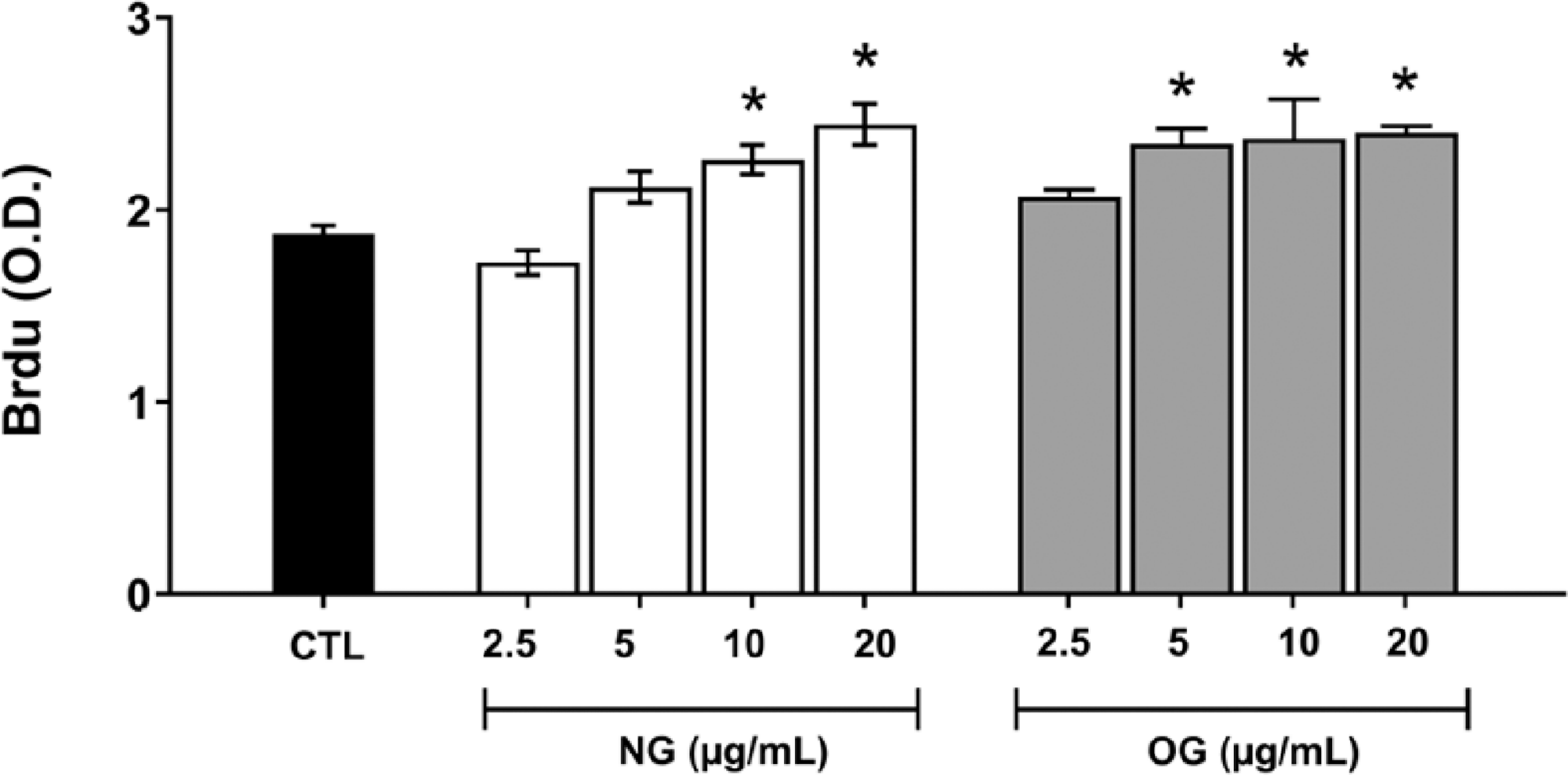
Proliferative effect on fibroblast cultures evaluated by the BrdU assay for nanoemulsion (NG) and essential oil (OG) at concentrations ranging from 2.5 to 20 μg/mL. Values are expressed as mean ± SD. The asterisk (*) Indicates a statistically significant difference compared to the control group (*p* < 0.05). The control (CTL) was used as a reference for data normalization.

#### Cell Migration (Scratch Assay)

The results of the cell migration assay demonstrated that both the NG and OG, at a concentration of 20 μg/mL, significantly reduced the wound area compared to the control group (untreated cells) after 16 h (Figure 9). Representative images of the artificial wound in the fibroblast monolayer at baseline (0 h) and after 16 h illustrate the reduction in wound area over time, highlighting the positive effect of both formulations in promoting cell migration

**Figure 9:**
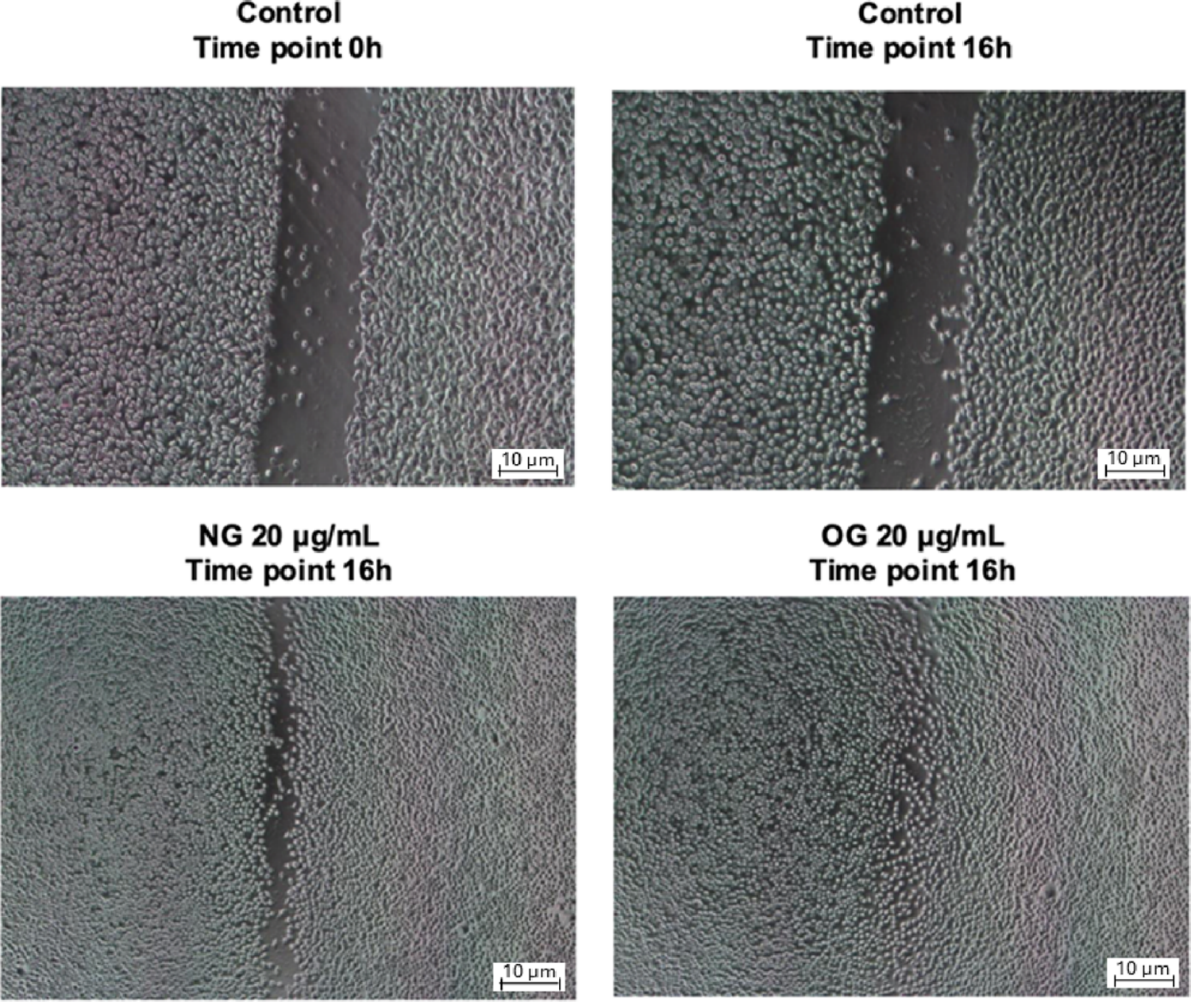
Representative images showing the effects of *Ocimum gratissimum* nanoemulsion (NG) and essential oil (OG) at a concentration of 20 μg/mL on fibroblast cell migration after 16 h compared to the control group at baseline (0 h).

### Tyrosinase Inhibition Assay

The results obtained from the tyrosinase inhibition assay with OG indicate that the enzyme’s inhibitory activity is concentration-dependent (Figure 10). Lower concentrations, such as 1 and 0.1 μg/mL, showed a marked decrease in inhibitory activity compared to the positive control (kojic acid at 125 μg/mL), reinforcing the direct relationship between essential oil concentration and its efficacy.

**Figure 10:**
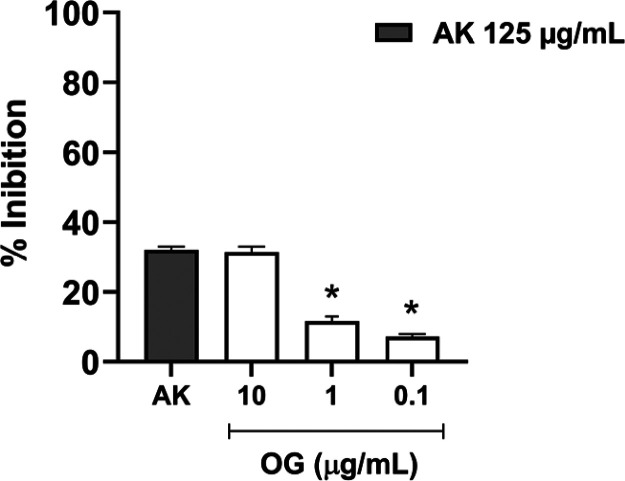
Percentage of tyrosinase inhibition by *Ocimum gratissimum* essential oil (OG) at different concentrations (10, 1, and 0.1 μg/mL) compared with kojic acid (KA, 125 μg/mL, positive control). Values are expressed as mean ± SD. The asterisk (*) Indicates a statistically significant difference compared to the KA control group (*p* < 0.05).

## Discussion

The results obtained in this study highlight the promising potential of the nanoemulsion containing *Ocimum gratissimum* essential oil as a biotechnological strategy to overcome limitations associated with the instability and low bioavailability of lipophilic bioactive compounds in cosmetic formulations. The chemical characterization revealed a phytochemical profile that differs from most studies involving OG in which eugenol is predominant.^8,9,13^ In this case, the abundance of β-ocimene and germacrene D highlights the influence of genetic, seasonal, and extraction method factors on the chemical composition of essential oil, factors often underestimated but crucial for the standardization and reproducibility of formulations.^42^

The physicochemical characterization of the nanoemulsion indicated the formation of a stable colloidal system, with particles within the ideal size range for topical application (10–500 nm), low polydispersity index (PDI), and negative zeta potential, parameters that enhance dermal absorption and enable the controlled release of bioactive compounds.^43,44^

The observed stability can be explained by the combination of small droplet size (<120 nm), narrow size distribution (PDI < 0.3), and steric stabilization by polysorbate 80, which minimizes coalescence and flocculation. As essential oils contain partially water-soluble constituents that can promote Ostwald ripening, uniform nanodroplets and storage at 4 °C were critical to slowing molecular diffusion, reflected in the absence of significant changes in droplet size and PDI over 60 days.^45,46^ This period was part of a preliminary assessment aimed at selecting the most promising formulation for subsequent biological assays, according to ANVISA’s Cosmetic Products Stability Guide, in which short-term results are used to screen candidates before long-term testing.^29^

These findings are consistent with those reported by Ontao et al.,^28^ with similar results observed in the physicochemical characteristics of the *Ocimum gratissimum* leaf oil nanoemulsion. In both studies, storage at low temperature (4 °C) was associated with better colloidal stability, resulting in either a decrease or minimal variation in particle size, which is attributed to the suppression of destabilization mechanisms, such as coalescence and Ostwald ripening. In contrast, storage at an elevated temperature (45 °C) led to an increase in droplet size, reinforcing the temperature sensitivity of the system.

The abrupt decrease in *Z*-average at 45 °C on day 60 is likely an artifact of intensity-weighted DLS analysis under thermal stress, where micellar solubilization of essential oil components and depletion of larger droplets leave smaller ones dominating the scattering signal.^47^ The slightly negative zeta potential values (−2 to −5 mV) result from the adsorption of nonionic surfactant molecules at the oil–water interface, exposing polar groups that acquire a small negative charge in aqueous medium. Although values above |± 30 mV| are often linked to electrostatic stabilization, here stability was predominantly due to steric effects from hydrated polysorbate 80 chains.^48^

The results reinforce that *O. gratissimum* nanoemulsions maintain optimal physical characteristics under refrigeration, as demonstrated by Ontao^28^ who reported greater colloidal stability and minimal changes in droplet size at lower temperatures.

Although antioxidant activity was not enhanced after nanoencapsulation, its maintenance is relevant considering the susceptibility of phenolic compounds to oxidative degradation in conventional formulations. This ensures prolonged functionality and protection against oxidative stress in topical applications, essential in products aimed at preventing skin aging and chronic inflammation.^49^

The significant reduction of key proinflammatory cytokines (TNF-α, IL-6, and IL-1) observed with the nanoemulsion (NG) highlights its potential to modulate inflammatory pathways. These biological effects are largely attributed to the presence of eugenol, a major component of OG known for its dual antioxidant and anti-inflammatory activities. Although eugenol is widely recognized for its anti-inflammatory activity, it was not the main constituent in the OG essential oil studied here. Therefore, the observed anti-inflammatory effects may result from synergistic interactions between eugenol and β-ocimene—the major constituent—as well as other minor terpenes. Essential oils are complex multicomponent mixtures, and their bioactivity often arises from the combined action of multiple constituents rather than a single compound.^51^

As described by Barboza,^50^ eugenol acts by scavenging reactive oxygen species (ROS), reducing lipid peroxidation, and inhibiting the production of inflammatory mediators such as nitric oxide, TNF-α, and IL-6 mechanisms, which supports its application in topical products targeting oxidative damage and inflammation. In addition to the intrinsic properties of eugenol, the use of a nanostructured delivery system enhances the performance of the formulation by promoting controlled release and improving skin permeation. This may explain the superior and prolonged effects of NG compared to the free essential oil, in agreement with previous studies that have demonstrated increased efficacy through nanoencapsulation strategies.^52^

The *in vitro* stimulation of fibroblast proliferation and migration further reinforces the wound healing potential of the formulation with wound closure times shorter than those reported in the literature for other plant extracts. This effect can be attributed to the action of phenolic compounds and terpenes capable of modulating the gene expression of factors involved in tissue regeneration and extracellular matrix synthesis.^39^

Innovatively, this study revealed the depigmenting potential of OG, with tyrosinase inhibitory activity comparable to that of kojic acid. This finding positions OG as an attractive natural alternative in skin-lightening formulations, especially in a market increasingly seeking to replace potentially irritating synthetic actives with safer and more sustainable options.^53^

Finally, the safety tests confirmed a favorable profile for the formulation despite the moderate irritation observed in the HET-CAM assay, indicating the need for adjustments in the emulsifier concentration. Surfactants such as polysorbate 80, although effective in stabilizing nanoemulsions, can compromise the skin barrier and induce irritation, highlighting the importance of balancing efficacy and tolerability.^54^ The phototoxicity evaluation on 3T3 fibroblasts, unprecedented for this nanoemulsion, revealed no relevant toxic effects following UV exposure, corroborating previous data on the safety of essential oil-based nanoemulsions. These findings support the safe and effective use of the formulation in cosmetic and therapeutic applications. However, adjustments in composition and additional stability studies are recommended to enhance its topical performance and technological feasibility.

## Conclusions

For the first time, this study employed an integrative methodological approach to validate the application of nanoencapsulated *Ocimum gratissimum* essential oil as a novel cosmetic active ingredient aligned with the principles of green cosmetics and biotechnology. The nanoemulsion (NG) formulation effectively enhanced the biological activity and stability of the essential oil, demonstrating significant antioxidant, anti-inflammatory, wound healing, and depigmenting properties.

The formulation exhibited robust physicochemical stability, an adequate safety profile, and superior bioactivity in comparison to the nonencapsulated essential oil across various *in vitro* assays. Its impact on key cellular parameters associated with oxidative stress, inflammation, and tissue regeneration supports its potential therapeutic application in cosmetic products, particularly those targeting skin barrier repair, hyperpigmentation reduction, and antiaging. Moreover, safety assessments confirmed an acceptable toxicological profile, with minor adjustments in the emulsifier concentration recommended to improve dermal tolerability.

## Supplementary Material



## Notes

Visual appearance of the nanoemulsion containing *Ocimum gratissimum* essential oil at a concentration of 1% (Figure S1) and visual appearance of the nanoemulsion containing *Ocimum gratissimum* essential oil before (D0) and after 60 days (D60) of preliminary stability testing under storage at 4, 25, and 45 °C (Figure S2) (PDF)

## ABBREVIATIONS

ABTS: 2,2’-azino-bis­(3-ethylbenzothiazoline-6-sulfonic acid
CAM: chorioallantoic membrane
CAM-TBS: chorioallantoic membrane-trypan blue staining
CPZ: chlorpromazine
CTL: control
DLS: dynamic light scattering
DPPH: 2,2-diphenyl-1-picrylhydrazyl
GC–MS: gas chromatography–mass spectrometry
H_2_O_2_: hydrogen peroxide
HET-CAM: hen’s egg test chorioallantoic membrane
L929: fibroblast cell line
LPS: lipopolysaccharide
MTT: 3-(4,5-dimethylthiazol-2-yl)-2,5-diphenyltetrazolium bromide
NG: nanoemulsion with *Ocimum gratissimum* essential oil
NO: nitric oxide
NRU: neutral red dye
OECD: Organization for Economic Co-operation and Development
OG: essential oil of *Ocimum gratissimum*
PDI: polydispersity index
RAW 264.7: macrophage cell line
RBC: red blood cell
