# Supplementary material for: Nanoemulsification Enhances the Regenerative and Safety Profile of Ocimum gratissimum Essential Oil In Vitro
Source: ACS Omega. 2025 Sep 28;10(39):45596–607. doi: 10.1021/acsomega.5c05769 (PMC12509127; doi:10.1021/acsomega.5c05769)
Supplement: Supplementary file 1 [file ao5c05769_si_001.pdf]

Nanoemulsification enhances the regenerative and safety profile of *Ocimum gratissimum* essential oil *in vitro*

Julia Salles Gava[a] *juliasallesgv@gmail.com* - https://orcid.org/0009-0005-0028-7771
Gabriela Aquino Simões[a] *gabriela-a-simoes@hotmail.com* - https://orcid.org/0009-0002-6838-9844
Danielle Braga Portes[a] *daniellebragaportes@gmail.com* - https://orcid.org/0000-0002-8639-5239
Juliana Varela Cruz[b] *julianacruz@usp.br* - https://orcid.org/0000-0001-6714-6483
Adriana Solange Maddaleno[c] *adrianamaddaleno@ub.edu* - *https://orcid.org/0000-0002-2049-7312*
Maria Pilar Vinardell[c] *mpvinardellmh@ub.edu* - *https://orcid.org/0000-0003-4533-5114*
Montserrat Mitjans[c] *montsemitjans@ub.edu* - https://orcid.org/0000-0003-1121-3581
Hildegardo Seibert França[d] *hildegardo.franca@ifes.edu.br* - *https://orcid.org/0000-0001-6129-8793*
Marcio Fronza[a]* *marcio.fronza@uvv.br* - *https://orcid.org/0000-0002-7316-8598*

[a]Vila Velha University, Natural Products Laboratory, Av. Comissário José Dantas de Melo, 21, 29102-920, Vila Velha, Brazil.

[b]São Paulo University, School of Pharmaceutical Sciences of Ribeirão Preto, Av. Bandeirantes, 14040-900, São Paulo, Brazil.

[c]Universitat de Barcelona, Department of Biochemistry and Physiology, Av. Joan XXIII, 27-31, 08028, Barcelona, Spain.

[d]Federal Institute of Espírito Santo, Bioproducts Development Laboratory, Av. Min. Salgado Filho, 29106-010, Vila Velha, Brazil.

***Corresponding author**

Prof. Dr. Marcio Fronza
Programa de Pós-Graduação em Ciências Farmacêuticas, Laboratório de Produtos Naturais, Universidade Vila Velha.
Av. Comissário José Dantas de Melo, n°21, Boa Vista, Vila Velha, ES, 29102-920, Brazil
E-mail: marcio.fronza@uvv.br
Telephone: +55 (27) 3421-2087

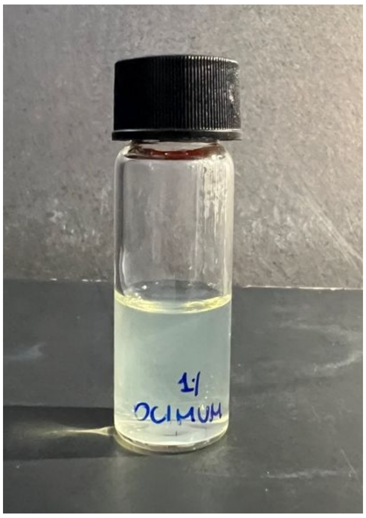

Figure S1. Visual appearance of the nanoemulsion containing *Ocimum gratissimum* essential oil at a concentration of 1%.

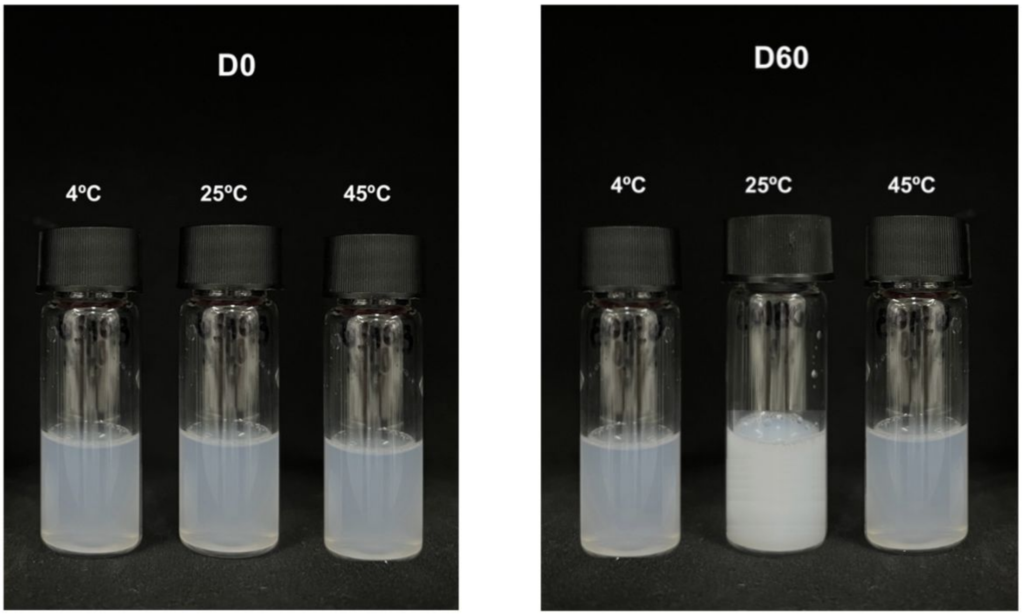

Figure S2. Visual appearance of the nanoemulsion containing *Ocimum gratissimum* essential oil before (D0) and after 60 days (D60) of preliminary stability testing under storage at 4 °C, 25 °C, and 45 °C.
